# Involvement of Dmp1 in the Precise Regulation of Hair Bundle Formation in the Developing Cochlea

**DOI:** 10.3390/biology12040625

**Published:** 2023-04-20

**Authors:** Yanmei Wang, Jihan Lyu, Xiaoqing Qian, Binjun Chen, Haojie Sun, Wenwei Luo, Fanglu Chi, Hongzhe Li, Dongdong Ren

**Affiliations:** 1 ENT Institute and Department of Otorhinolaryngology, Eye & ENT Hospital, Fudan University, Shanghai 200031, China; 2 NHC Key Laboratory of Hearing Medicine, Fudan University, Shanghai 200031, China; 3 Department of Otolaryngology-Head and Neck Surgery, Guangdong Provincial People’s Hospital, Guangdong Academy of Medical Sciences, Guangzhou 510080, China; 4 The Second School of Clinical Medicine, South Medical University, Guangzhou 510080, China; 5 Research Service, VA Loma Linda Healthcare System, Loma Linda, CA 92350, USA; 6 Department of Otolaryngology-Head and Neck Surgery, Loma Linda University School of Medicine, Loma Linda, CA 92350, USA

**Keywords:** Dmp1, inner ear development, cell-intrinsic polarity, stereocilia morphology, kinocilium positioning

## Abstract

**Simple Summary:**

Deafness is a common clinical disease and a major global public health problem worldwide. The hair cell regeneration is one of the most promising strategies to address hearing loss. Thus, it is urgent to explore the development and differentiation process of hair cells at the cellular and molecular level, which is the basis of “overall regeneration of functional auditory receptors”. We observed the characteristics of Dmp1 involved in inner ear morphogenesis by using recombinant enzyme (Cre) transgenic mice to knockdown Dmp1 in early inner ear development. In our study, we found Dmp1 was already expressed early in inner ear development and Dmp1 mutant mice had abnormal hair cell morphology (disordered stereocilia and mislocalized kinococilia). It is reasonable to think that Dmp1 plays a role in the precise regulation of hair bundle morphogenesis. Dmp1 may be a suitable target molecule for the treatment of deafness, and our study provides strong support for gene therapy for sensorineural deafness.

**Abstract:**

Dentin matrix protein 1 (Dmp1) is a highly phosphorylated, extracellular matrix protein that is extensively expressed in bone and teeth but also found in soft tissues, including brain and muscle. However, the functions of Dmp1 in the mice cochlea are unknown. Our study showed that Dmp1 was expressed in auditory hair cells (HCs), with the role of Dmp1 in those cells identified using Dmp1 cKD mice. Immunostaining and scanning electron microscopy of the cochlea at P1 revealed that Dmp1 deficiency in mice resulted in an abnormal stereociliary bundle morphology and the mispositioning of the kinocilium. The following experiments further demonstrated that the cell-intrinsic polarity of HCs was affected without apparent effect on the tissue planer polarity, based on the observation that the asymmetric distribution of Vangl2 was unchanged whereas the Gαi3 expression domain was enlarged and Par6b expression was slightly altered. Then, the possible molecular mechanisms of Dmp1 involvement in inner ear development were explored via RNA-seq analysis. The study suggested that the Fgf23–Klotho endocrine axis may play a novel role in the inner ear and Dmp1 may regulate the kinocilium–stereocilia interaction via Fgf23–Klotho signaling. Together, our results proved the critical role of Dmp1 in the precise regulation of hair bundle morphogenesis in the early development of HCs.

## 1. Introduction

Sensorineural hearing loss (SHL) is the most prevalent sensory deficit in humans, with about 1 in 1000 children born with significant hearing impairment [1]. Approximately 50% of all patients with congenital hearing impairment are thought to have a genetic etiology [2,3]. Although more than 200 different hearing-related genes have been identified to date, a far larger number is expected [4]. Therefore, further efforts, including the use of animal models, are needed to identify those genes involved in cochlear development. 

The organ of Corti (OC), located within the cochlea, has a sophisticated architecture, with two types of sensory hair cells (HCs) arranged as three rows of outer hair cells (OHCs) and one row of inner hair cells (IHCs) [5]. Interdigitating between these HCs are intervening non-sensory support cells, which form an intricate checkerboard pattern that extends along the longitudinal axis of the cochlea [6]. At the cellular level, hundreds of actin-based stereocilia arrange in graded heights into a bilaterally symmetrical V-shaped pattern and a single microtubule-based kinocilium is found at the vertex of the V-shaped bundles on the apical surface of each HC. Therefore, this proper positioning of the kinocilium together with the structural asymmetry of the stereociliary bundle defines the cell-intrinsic polarity of HCs. On the other hand, the vertices of the stereocilia uniformly point toward the abneural edge of the cochlear duct, thus establishing planar cell polarity (PCP) at the tissue level [7,8]. Yet little is known about the mechanism of hair bundle development and PCP establishment.

Dentin matrix phosphoprotein 1 (Dmp1), a member of the small integrin-binding ligand, N-linked glycoprotein (SIBLING) superfamily, is an acidic, non-collagenous, extracellular matrix protein [9]. It was originally discovered in a rat incisor cDNA library and subsequently shown to be mainly expressed in bone and dentin [10,11]. It is a key regulator that promotes both biomineralization and serum phosphate homeostasis [12]. However, it has also been detected in soft tissues (brain, muscles, liver, pancreas and kidney) and tumors [13], suggesting multiple functions beyond maintaining mineral homeostasis. For example, Dmp1 is highly expressed at the blood–brain barrier and is a novel element related to its integrity and astrocyte maturation [14]. It is also upregulated in prostate neoplasms and plays a key role in cancer progression by colocalizing and interacting with its specific cognate, Mmp9 [15]. Clinically, *DMP1* mutation has been shown to result in autosomal recessive hypophosphatemic rickets (ARHP), and one case study described a family with ARHP; owing to a novel homozygous *DMP1* mutation, two probands had hearing deficit [16]. Several previous clinical studies have also reported sensorineural deafness associated with recessive hypophosphataemic rickets [17,18,19,20]. The hearing loss occured in almost all adult patients and some children had progressive hearing loss beginning later in life. In addition, Dentin Sialophosphoprotein (DSPP), another member of the SIBLING proteins (DSPP and DMP1 share many similarities in both their gene and protein structures), is from a known deafness gene (*DFNA39*) that can cause congenital sensorineural deafness [21,22,23].

It is thus conceivable that Dmp1 may play a role in the cochlea development. To verify this possibility, we observed the OC of Dmp1 conditional knockdown mice during their postnatal development. In doing so, we discovered a potential role of Dmp1 in regulating the morphogenesis of the auditory hair bundles in the developing cochlea.

## 2. Materials and Methods

### 2.1. Mice

All animal trials were authorized by the Animal Care and Use Committee of the EENT Hospital of Fudan University, Shanghai. Dmp1^flox/flox^ mouse mutants were provided by Sun Yao (Tongji University); their generation and genotyping were previously described [24]. Foxg1 Cre mice (C57BL/6J and BALB/C mixed background) were kindly provided by Li Huawei (Fudan University). Cre was knocked in at the Foxg1 locus and the Foxg1-Cre mice were crossed with Dmp1^flox/flox^ mice to obtain Dmp1^flox/+^:Foxg1-Cre+ mice [25]. Dmp1 conditional knockdown (cKD) mice in the inner ear were generated by crossing Dmp1^flox/+^:Foxg1-Cre+ mice with Dmp1^flox/flox^ mice to generate Dmp1^flox/flox^:Foxg1-Cre+ mice. The primers for genotyping were as follows: Dmp1-A1loxP-F:5′-GCAGGTTGTAGCACTGAGGA-3′ and Dmp1-A2loxP-R:5′-CTTTGACAGTGTCTTATCCAATAGC-3′ (with the expected sizes of the amplicons being Dmp1^flox/flox^-156 bp and Dmp1 wild type (WT)-212 bp), and GA486:5′-TGCCACGACCAAGTGACAGCAATG-3′ and GA487:5′-ACCAGAGACGGAAATCCATCGCTC-3′ (with the expected size of the Cre allele being 300–400 bp).

### 2.2. Immunohistochemistry

Dissected otic capsules from mice at P1 were fixed in 4% paraformaldehyde at 4 °C for 2 h. Then, the cochleae were dissected from the otic capsules and immunohistochemically analyzed as previously described [26]. Unless otherwise noted, all imaging pictures were taken from the mid-basal region of the cochlea. The following primary antibodies were used: anti-Dmp1 (1:100; Abcam, Cambridge, UK), anti-Gαi3 (1:200; Sigma, St Louis, MO, USA), anti-Par6b (1:200; Santa Cruz Biotechnology, Santa Cruz, CA, USA), anti-ZO-1 (1:200; Santa Cruz Biotechnology, Santa Cruz, CA, USA), anti-β-spectrin (1:200; BD Biosciences, San Jose, CA, USA), anti-β-catenin (1:100; Cell Signaling, Danvars, MA, USA), anti-α-tubulin (1:200; Sigma, St Louis, MO, USA), anti-Cdh23 (1:200; Abcam, Cambridge, UK) and anti-Vangl2 (1:200; R&D Systems, Minneapolis, UK). An LSM510 laser confocal microscope (Carl Zeiss, Oberkochen, Germany) was used to observe the specimens at 63× magnification with excitation wavelengths of 647, 555 and 488 nm. The scanning aperture was 1 unit, the linear average was 4 times, the scanning speed was 7 and the image resolution was 1024 × 1024. Confocal microscopy images were z-stacks of all planes in which the proteins expressed and were processed with Adobe Photoshop.

### 2.3. Electron Microscopy

Cochlear specimens were dissected and fixed with 2.5% glutaraldehyde overnight at 4 °C and processed in 1% osmic acid, followed by dehydration in a graded ethanol series. This was followed by critical point drying in a chilled Polaron E3000 Critical Point Dryer (Quorum Tech, Laughton, UK), and gold coating for 3.5 min. Scanning electron microscopy (SEM) images were obtained by using a field emission scanning electron microscope (DS-130F; Topcon, Japan) at 10 kV.

### 2.4. Quantitative qPCR

RNA was extracted from the cochlear basilar membrane of P1 Dmp1 cKD and WT mice using the Trizol-based RNA extraction method and total RNA was reverse transcribed to cDNA using Superscript III reverse transcriptase (Invitrogen, Carlsbad, CA, USA) based on the recommendations provided by manufacturer. Quantitative PCR (qPCR) was carried out using a TB Green™ Prime Script™ RT-PCR kit (Takara) on an ABI 7500 real-time PCR system (Applied Biosystems, Waltham, MA, USA). The extracted total RNA was 100~150 ng/µL, and the amount of cDNA used in the qPCR reaction system (20 µL) was 40~60 ng. The qPCR reaction parameters were the following: 94 °C for 2 min, and 40 cycles of 94 °C for 5 s, 60 °C for 30 s, 72 °C for 30 s and a final extension at 72 °C for 2 min. Samples were each run in triplicate. Relative expression was normalized to the level of the housekeeping gene Gapdh and calculated using the 2^−ΔΔCt^ method. Specific primer sets are listed in Table 1.

### 2.5. Western Blotting

Cochlear tissues from Dmp1 cKD and WT were lysed with ice-cold RIPA lysis buffer (UBI). Then, the lysate was centrifuged at 12,000 rpm for 15 min at 4 °C. Next, we transferred the supernatant to an EP tube. The protein concentration was determined using a BCA kit (UBI); the concentration was 1.5–2.3 µg/µL. We diluted the supernatant to 1.5 µg/µL with a loading buffer. The sample was denatured at 95 °C for 10 min. The loading amount was 15 µg/loading. The supernatant was subjected to SDS-PAGE and the resulting protein bands were electroblotted onto a PVDF membrane. The membrane was incubated for 1 h in TBST containing 5% skim milk powder to block unspecific binding. Then, it was incubated with primary antibodies (Dmp1, 1:1000, Bioss; Gapdh, 1:5000, Abcam) overnight at 4 °C, and HRP-conjugated secondary antibody (mouse (1:5000, Abcam) and rabbit (1:5000, Abcam)) for 2 h at room temperature. Dmp1 was monitored using an anti-Dmp1 rabbit antibody. The signals were detected using a gel imaging system (Clinx) for exposure imaging.

### 2.6. RNA Sequencing and Bioinformatic Analysis

Total RNA extracted from the cochlea of Dmp1 cKD and WT mice at P1 was subjected to RNA-seq assays and the specific procedures were referred to in our previously published article [26]. Differentially expressed genes (DEGs) were identified using Cuffdiff, with a significant difference based on a Bonferroni-corrected value of <0.05. Then, GO annotation and KEGG pathway analyses were performed for the resulting DEGs using the DAVID database. The TXT results files were preserved for subsequent analysis. Moreover, GO and KEGG data were visualized using Bioinformatics (http://www.bioinformatics.com.cn, accessed on 12 January 2022). STRING was used to analyze the protein–protein interaction network of the DEGs and Cytoscape software (v3.7.1) was used to establish a gene coexpression network and screen for hub genes.

### 2.7. Phenotypic and Statistical Analysis

All images were postprocessed in Adobe Photoshop (San Jose, CA, USA). Areas of Gαi3 expression were measured using ImageJ software. Differences between group means were analyzed using an unpaired non-parametric *t*-test (Mann–Whitney U test) and a *p* value < 0.05 was considered indicative of statistical significance. Data were plotted and analyzed statistically using GraphPad Prism6 (San Diego, CA, USA). 

## 3. Results

### 3.1. Dmp1 Expression in Developing HCs and Conditional Inactivation of Dmp1 in the Cochlea

**Figure 1:**
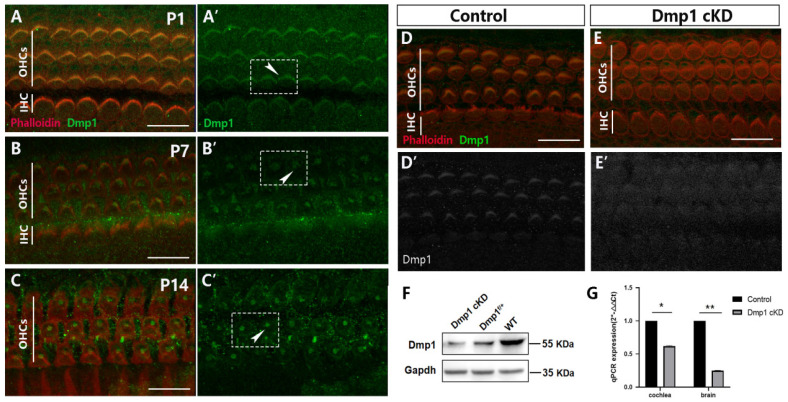
Dmp1 was dynamically expressed in the developing cochlea and the signal of Dmp1 was significantly weaker in mutant mice. (**A**–**C**): Confocal images of HCs in the mid-basal regions stained. Dmp1 staining (green) was singly showed in (**A′**–**C′**). Cell boundaries and hair bundles were labeled by using phalloidin staining (red). At P1, the Dmp1 expression coincided with the hair cell stereocilia bundles (arrowhead in **A′**). At P7 and P14, Dmp1 was gradually concentrated at the V-shaped apex of stereocilia and became a cloud cluster (arrowheads in **B′**,**C′**). (**D**,**E**): Efficiency of Dmp1 deletion shown by mid-basal regions in P1 cochlea of Dmp1 cKD and control mice, Dmp1 staining (gray) was singly showed in (**D′**,**E′**). Dmp1 was still detected in HCs of Dmp1 cKD with a much lower signal than in the control. (**F**): Western blot analysis of Dmp1 protein expression. Gapdh was used as the reference protein. It was shown that Dmp1 protein expression (gray band) was significantly lower than Dmp1^flox/+^ and WT mice. (**G**): Quantitative analysis of Dmp1 at RNA level by qPCR. Dmp1 RNA expression was significantly reduced in the cochlea and brain tissues of mutant mice. Error bars represented the standard deviation and the statistical significance was assessed using a *t*-test. * *p* < 0.05; ** *p* < 0.01 (n = 3 each). Scale bars: 10 µm. (See Figures S4–S9 for original Western blot images).

The subcellular localization of Dmp1 in cochlear HCs was determined by immunostaining using an anti-Dmp1 antibody. Stereocilia and the actin cytoskeleton were visualized using phalloidin. At P1, P7 and P14, Dmp1 was detected in almost all cochlear HCs of WT mice, with the expression location changing depending on the developmental stage (Figure 1A–C). At P1, the site of Dmp1 expression coincided with the stereociliary bundles of the HCs. By P14, Dmp1 had concentrated at the V-shaped apex of the stereocilia, forming a cloud-like cluster. The pattern of Dmp1 expression indicated that the protein played a role in cochlear HC development.

To explore the potential functional involvement of Dmp1 during HC development, Dmp1 in the cochlear epithelium was conditionally knocked down by breeding mice carrying the Dmp1-floxed allele with mice harboring a Cre recombinase at the locus of Foxg1, which expressed Cre restrictedly in the developing telencephalon and discrete head structures starting at P8.5 [25]. Hereafter, these mice are referred to as Dmp1 cKD mice. The Dmp1 cKD mice were alive and fertile and their overall morphology was indistinguishable from that of WT mice. However, Dmp1 expression in the knockdown mice was significantly weaker than in the WT mice, as determined by whole-mount immunostaining of the cochlear basilar membrane (Figure 1D,E). Western blotting and qPCR analyses showed that Dmp1 mRNA and protein levels were suppressed in the brain and cochlear tissue of the mutant mice (Figure 1F,G).

### 3.2. Dmp1 Deficiency Leads to Stereociliary Bundle Deformity but Not the Loss of HCs

**Figure 2:**
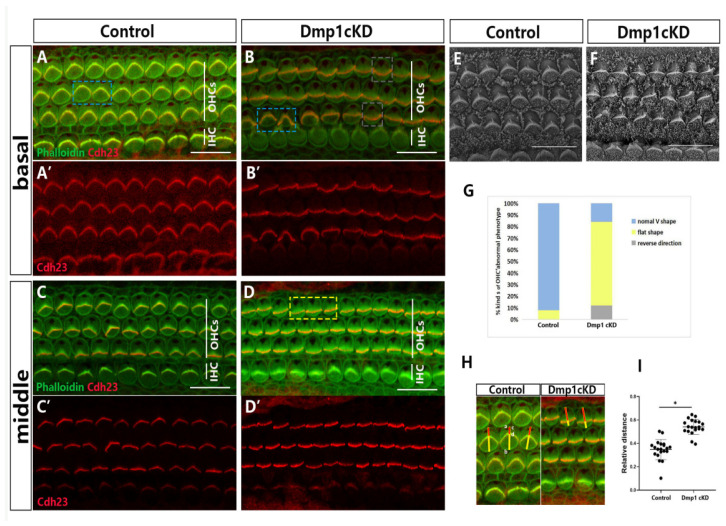
Stereocilia hair bundles defected in Dmp1 cKD mice. (**A**–**D**): Whole-mount images of middle and basal turns of cochlea from control and Dmp1 cKD mice, Cdh23 staining of stereocilia junction protein was singly showed in (**A′**–**D′**). In the control cochlea (**A**,**C**), the stereocilia bundles were “V”-shaped and essentially symmetrical (blue bracket), while many hair cells’ stereocilia bundles had a defective shape such as a flat shape (yellow bracket) and reverse direction (grey bracket) in Dmp1 cKD cochlea (**B**,**D**). (**E**,**F**): SEM of hair cells from basal cochlear turns of Dmp1 cKD and control mice at P1. (**G**): Stacked bar chart of ratio of kinds of OHC hair bundles phenotype in knockdown and control mice (3 mice per group). (**H**,**I**): The amesiality of hair bundles in Dmp1 cKD mice. As shown in (**H**), the stereocilia bundles were globally moved toward one side (inner hair cells side) in single cells of Dmp1 cKD mice. It can be quantified by the ratio of cd/ab. ab (yellow line): the distance from the center of the blank area of the cuticular plate to the other side of the hair cell (close to the inner hair cell side). cd (red line): the center of the blank area to the V-shape vertex of hair bundles. Moreover, the result was shown as the cluster diagram in (**I**). Statistical significance was calculated using a *t*-test. * *p* < 0.05. Scale bars: 10 µm (**A**–**D**) and 5 µm (**E**,**F**).

The effect of Dmp1 deficiency in Dmp1 cKD mice on HC development was assessed by comparing the cochleae of Dmp1 cKD and WT mice at P1. At the gross level, there were no obvious differences in either the morphology of the otic vesicle or the size of the cochlear basilar membrane. Whole-mount staining of the OC showed the overall arrangement was normal and there was no absence of hair cells. Next, we examined the stereociliary morphology of the HCs at P1 by using phalloidin staining of the stereociliary F-actin core and Cdh23 staining of stereocilia junction protein, located at the stereociliary tip. In the middle and basal turns of WT cochlea, a normal orientation and morphology of the stereociliary bundles of the OHCs and IHCs was observed (Figure 2A,C). However, the OHCs of Dmp1 cKD mice were characterized by large numbers of dysmorphic hair bundles, including flattened, wavy and inverted shapes, usually with no clear vertices, but the stereociliary bundles of the IHCs were largely unaffected (Figure 2B,D). The percentage of flattened stereociliary bundles in the OHCs was significantly higher in Dmp1 cKD mice than in WT mice (76% vs. 4%; 91 flattened bundles of 121 hair cells in Dmp1 cKD mice and 4 of 125 in WT mice. Figure 2G). Electron microscopy scanning also revealed that the stereocilia in Dmp1 cKD mice were mostly of a flat shape (Figure 2E,F). This abnormal stereociliary bundle morphology indicated the altered cell-intrinsic polarity of the HCs of Dmp1 cKD mice. In addition, in the Dmp1 cKD mice, the stereociliary bundles were globally shifted towards the IHCs, as seen on the surface of single cells (Figure 2H,I), indicative of a change in the overall subcellular localization of the stereocilia. Together, these observations pointed to a role of Dmp1 in maintaining the normal V-shape of hair bundles during the early morphogenesis of auditory HCs. 

### 3.3. Misplaced or Occasionally Absent Kinocilia in Dmp1-Deficient Cochlea

**Figure 3:**
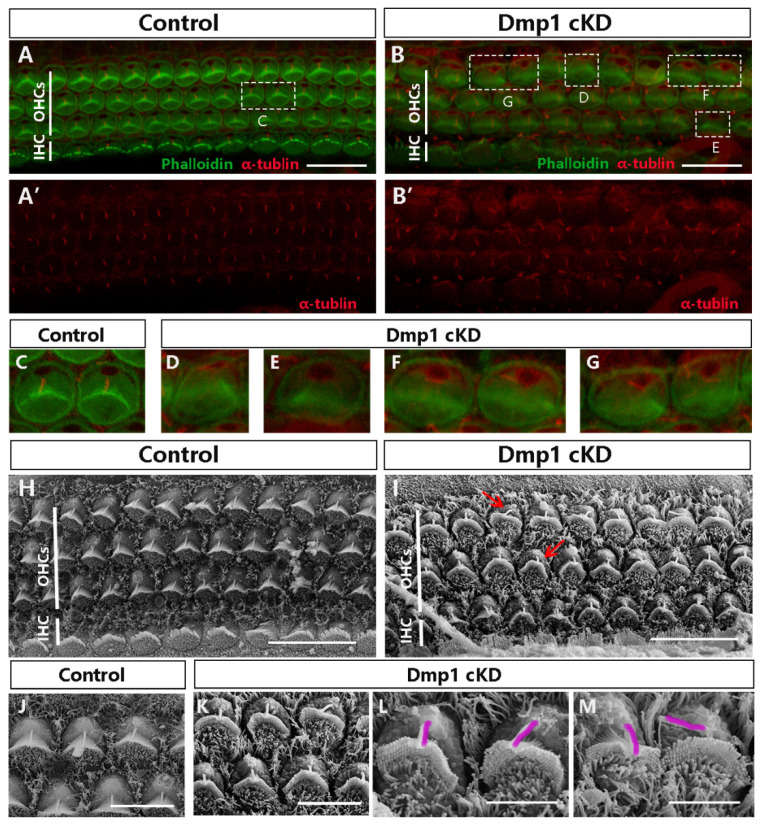
The proper positioning of kinocilia was affected in Dmp1 cKD mice. (**A**,**B**): Confocal microscope images from the basal regions of Dmp1 cKD and littermate control mice cochlea, Acetylated tubulin staining was singly showed in (**A′**,**B′**) (Acetylated tubulin: red. Phallodin: green.) (**C**–**G**): The amplification of the white dashed box in (**A**,**B**). In normal control cochlea, hair cells had a single kinocilium located at the vertex of the stereociliary bundles (**C**). By contrast, in the Dmp1 cKD mice, the kinocilia were misplaced and deviated from the vertex of the V-shape hair bundles, and the bundles were deformed, with no clear vertex (**D**,**F**,**G**). Several hair cells also had deletions of kinocilium (**E**). (**H**–**M**): SEM of outer hair cells from basal cochlear turns of Dmp1 cKD and control mice at P1 using scans with different magnifications. (**H**,**I**): 3 k magnification. (**J**,**K**): 8 k magnification. (**L**,**M**): 15 k magnification. Red arrows showed the abnormal positioning of kinocilia.Under the higher magnification, the separation between kinocilia and stereociliary bundles was clearly noted. The kinocilia were outlined (purple). Scale bars: (**A**–**I**): 10 µm; (**J**,**K**): 5 µm; and (**L**,**M**): 3 µm.

The kinocilia of hair cells are required for the polarity of the stereociliary bundles. Abnormal localization of kinocilia relative to hair bundles results in bundle malformations. Therefore, we examined whether the position of kinocilia was different in Dmp1-deficient HCs, using acetylated tubulin (α-tubulin) as a marker of kinocilia (Figure 3A–G). Normal kinocilia have a defined position and structural polarity; they are located at the vertex of the V-shaped stereociliary bundles and centered next to the tallest stereocilia (Figure 3A,C). However, in the Dmp1 mutants, the kinocilium was not properly positioned, as it was frequently separated from the stereociliary bundles, with a loss of the kinocilium seen in some HCs (Figure 3B,D–G). These findings were confirmed by using scanning electron microscopy (Figure 3H–M). The failure of the kinocilia to attach to the highest row of the stereocilia in the posterior part of the bundle suggested the uncoupling of the kinocilia from the hair bundles in the cochlea.

### 3.4. Dmp1 Deficiency Affects Intrinsic Cell Polarity Rather than Planar Cell Polarity

**Figure 4:**
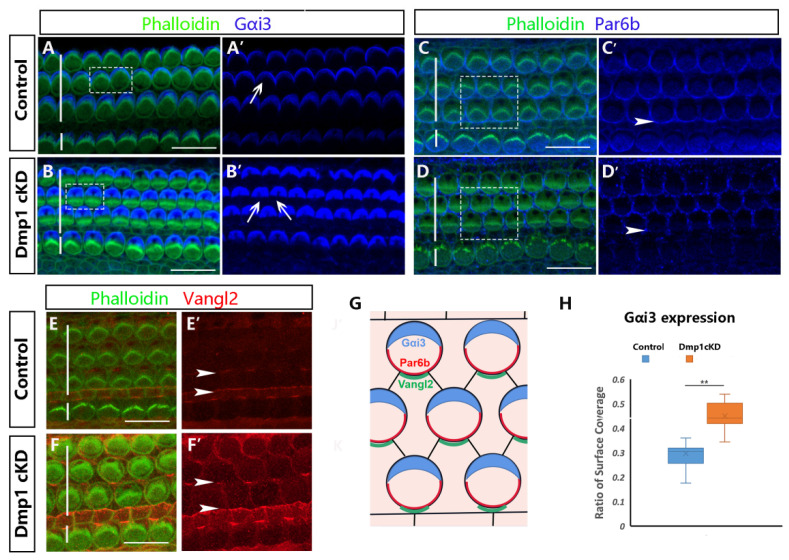
Dmp1 inactivation affected the cell-intrinsic polarity of HCs. (**A**–**D**): Gαi3 and Par6b localization in the control and Dmp1 cKD OC at P1, Gαi3 and Par6b staining was singly showed in (**A′**–**D′**). (**G**): Cartoon depicting asymmetric localization of Gαi3, Par6b and Vangl2 in the organ of Corti. It was obvious that Gαi3 formed a crescent-shaped region lateral to the stereocilia bundle on the apical surface of HCs and the region of Gαi3 protein expression was expanded significantly in mutant mice (white arrows in **B′**). Par6b is expressed on the medial apical surface and the localization of Par6b in the first row of hair cells was subtly altered and absent in mutant mice (white arrows in **D′**). (**H**): Box plot of the quantification of Gαi3 expression expansion (the ratio of Gαi3 region position to the total epidermal plate area), counting 125 OHCs from 3 WT mice and 121 OHCs from 3 Dmp1 cKD mice. (**E**,**F**): Localization of the core PCP components Vangl2 in control and Dmp1 cKD cochlea, Vangl2 staining was singly showed in (**E′**,**F′**). There was largely intact asymmetric localization of Vangl2 along the medial HC junctions.Statistical significance was calculated using a *t*-test. ** *p* < 0.01. Scale bars: 10 µm.

The core PCP proteins are important regulators of kinocilium orientation and mutation in core PCP components could cause aberrant positioning of the kinocilium, due to disrupted intercellular signaling. Thus, based on the abnormal ciliary morphology of OHCs in Dmp1 cKD mice, we aimed to explore the association between the Dmp1 and PCP pathways. The involvement of core PCP components in Dmp1 cKD mice was determined by staining cochlear whole mounts from P1 mice with antibody to Vang-like 2 (Vangl2), a core component of the PCP pathway (Figure 4E,F), and then comparing the distribution of Vangl2 in WT and Dmp1 cKD cochlea. In the normal cochlea, Vangl2 was expressed at the level of the adherens junction and localized asymmetrically at the junctions between the medial and lateral cell surfaces of HCs. A similar signal distribution of Vangl2 was seen in the cochlea of Dmp1 cKD mice, despite their abnormal hair bundles. This finding suggested that Dmp1 protein deficiency would not affect PCP signaling.

The organization of the stereociliary bundle and the location of the kinocilium reflect the cell-intrinsic polarity in the OC. We therefore examined cell-intrinsic polarity in Dmp1 cKD mice by immunostaining for the markers Gαi3 and Par6b, expressed on the lateral and medial apical surfaces of HCs, respectively. These proteins interact with the cortical cytoskeleton underlying the apical surface of HCs and their deletion disrupts the migration of the kinocilium at the surface, with consequent effects on the shape of the hair bundles. We observed an expansion of the Gαi3 expression domain on the surface of the HCs in Dmp1 cKD mice (Figure 4A,B,H) and subtle defects in Par6b expression in the first row of the OHCs (Figure 4C,D), consistent with the flattened morphology of the stereociliary bundles and kinociliary mislocalization. 

The localization of other proteins was also assessed, including β-catenin, a component of the adherens junction; ZO-1, a tight junction marker; and β-spectrin, an actin-associated protein (Figures in Supplementary Materials). All of these proteins were largely unaffected in Dmp1 cKD cochlea, suggesting that Dmp1 was not required for their recruitment to their cellular contacts or for normal epithelial apical–basal polarity. 

### 3.5. Transcriptomic Changes in Dmp1-Deficient Cochlea

**Figure 5:**
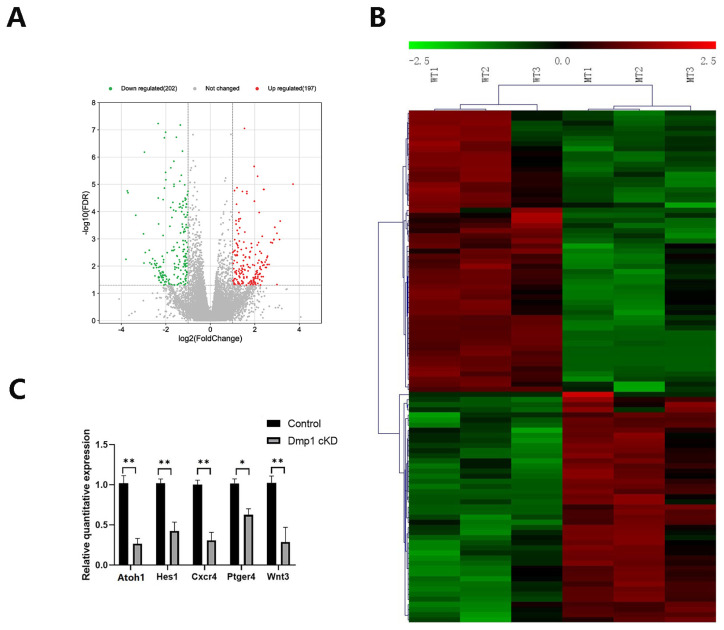
Transcriptomic change in Dmp1-deficient cochlea. (**A**): The volcano plots of all DEGs. Statistically, a gene was considered differentially expressed if the fold change > 2 and the adjusted *p* value < 0.05 (four dotted lines). Data points in red represent upregulated genes and green represent downregulated genes. (**B**): Heatmap generated by clustering 389 DEGs and 6 samples together. Red represents upregulated DEGs, while green represents downregulated DEGs. (**C**): qPCR validation of RNA-Seq results showed that the expressions of Atoh1, Hes1, Cxcr4, Ptger4 and Wnt3 were significantly reduced in Dmp1 cKD cochleae. Data were expressed as mean ± SEM. n = 3; using *t* test, * *p* < 0.05 and ** *p* < 0.01.

Insights into the molecular mechanism underlying the observed abnormal morphology of the HC bundles in Dmp1 cKD mice were obtained via RNA-seq transcriptome analysis. Inner ear samples of three WT and three Dmp1 cKD mice at P1 were analyzed, which resulted in an average of 30.2 ± 2.1 × 10^3^ transcripts per sample. Gene expression profiles of Dmp1 cKD and WT mice were compared using a volcano plot (Figure 5A). While the majority of transcripts were not differentially expressed between WT and Dmp1 cKD mice, 389 significant DEGs were identified (202 downregulated and 197 upregulated genes), based on a fold change > 2 and an FDR-corrected *p* value of <0.05. The top 30 up- or downregulated DEGs are shown in Table 2. Using cluster analysis, a heatmap showed that six samples clustered into two related groups based on similar expression patterns (Figure 5B). Five DEGs in the Dmp1 cKD cochlea (Atho1, Hes1, Cxcr4, Ptger4 and Wnt3) were analyzed by using qPCR, which confirmed the expression patterns determined by RNA-seq (Figure 5C).

**Figure 6:**
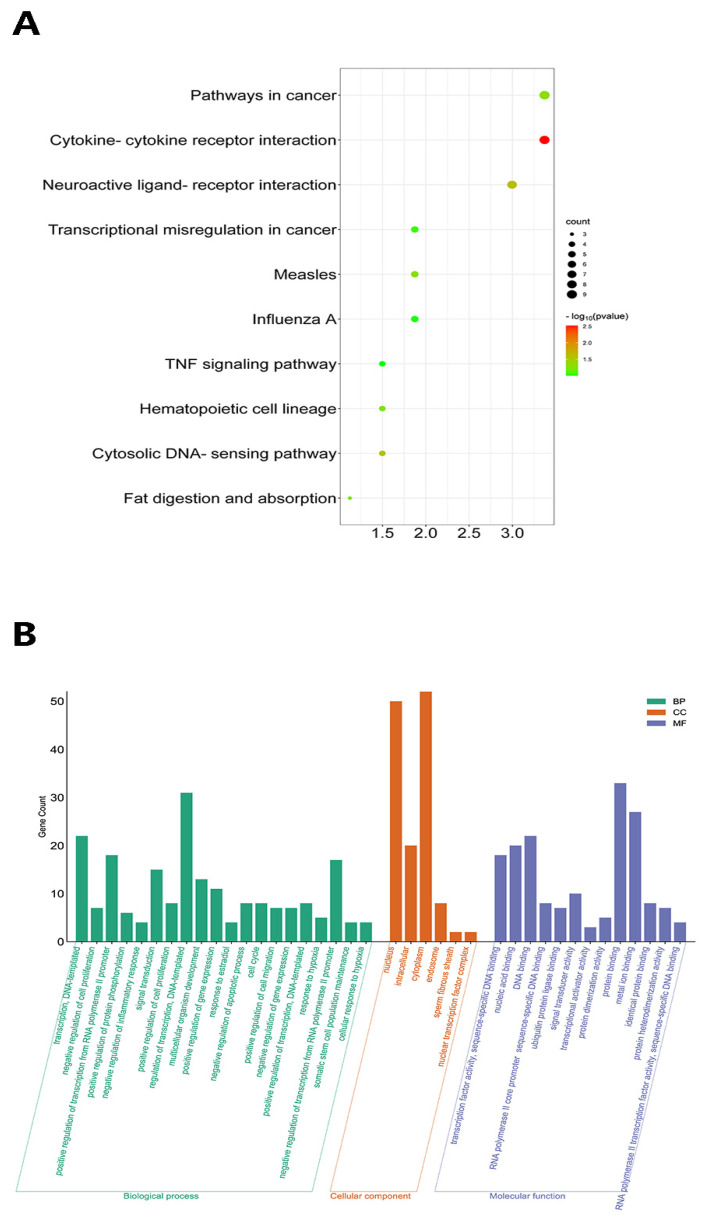
Visualization of GO and KEGG pathway enrichment analysis. (**A**): KEGG enrichment bubble plot. The color of the bubble indicates the *p*-value and the size indicates the number of genes. (**B**): GO enrichment significant bar chart. The names of gene ontology are seen in the horizontal axis and gene count in the vertical axis. The result of GO functional enrichment analysis is composed of 3 parts: cellular component (green bar), biological process (yellow bar) and molecular function (blue bar).

To investigate the biological processes directly related to these DEGs, GO and KEGG pathway analyses of this genes were performed. Three processes were identified in the GO analysis: biological process (BP), cellular component (CC) and molecular function (MF) (Figure 6B). Among the processes attributed to BP were the regulation of transcription from the RNA polymerase II promoter, GO:0006357 (15.03%); the negative regulation of transcription from the RNA polymerase II promoter, GO:0000122 (9.44%); signal transduction, GO:0007165 (9.44%); multicellular organism development, GO:0007275 (8.04%); and the positive regulation of cell proliferation, GO:0008284 (6.64%). The KEGG pathway analysis identified significantly enriched pathways associated with the DEGs, including Herpes simplex virus one infection, mmu05168 (8.39%); pathways in cancer, mmu05200 (4.54%); cytokine–cytokine receptor interaction mmu04060, (3.84%); HIF-1 signaling pathway, mmu04066 (3.54%); NF-kappa B signaling pathway, mmu04064 (2.12%); Toll-like receptor signaling pathway, mmu04620 (1.12%); and TNF signaling pathway, mmu04668 (3.25%) (Figure 6A).

**Figure 7:**
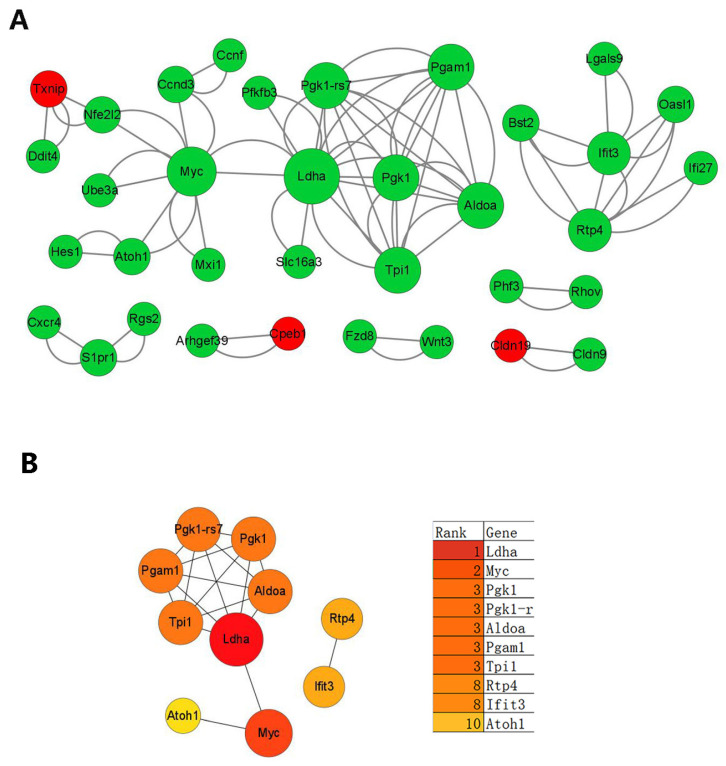
PPI network of DEGs. (**A**): Red represents upregulated genes while green represents downregulated genes and the nodes (proteins) with larger size have higher degree (number of interactions). (**B**): The top 10 hub genes are listed (color depth expressed degree height).

On the basis of the proteins encoded by the DEGs, a protein–protein interaction (PPI) network was constructed. Following the importation of the 389 DEGs into the STRING online database and visualization using the Cytoscape software, 38 nodes (34 upregulated and 4 downregulated) and 64 interaction pairs were generated (Figure 7A). The top 10 hub genes in the network were identified as Ldha, Myc, Pgk1, Pgk1-r, Aldoa, Pgam1, Tpi1, Rtp4, Ifit3 and Atoh1 (Figure 7B).

**Figure 8:**
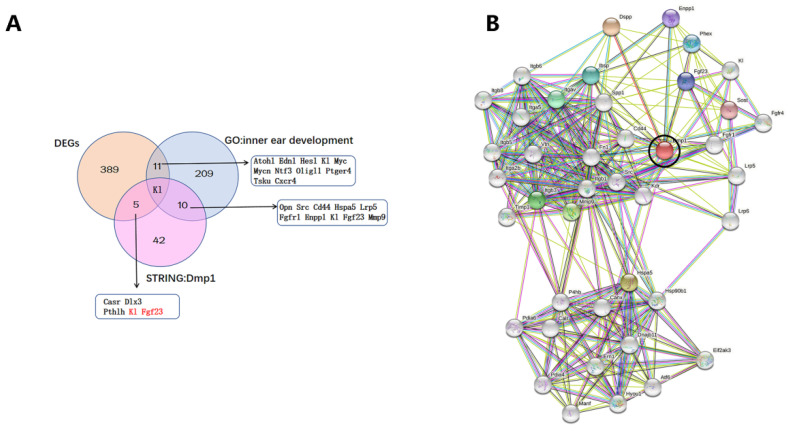
(**A**): Venn diagram among the GO: inner ear development, 389 DEGs and the Dmp1 STRING database. (**B**): STRING database proteins interacting with Dmp1 (black circle in (**B**)).

A PubMed search was conducted to identify target genes of the DEGs that were related to inner ear development. Lists of the proteins that interact with Dmp1 according to the STRING database (Figure 8B) were made and then compared to each other in a Venn diagram to identify target genes (Figure 8A). DEGs-encoded proteins that interact with Dmp1 were Casr, Dlx3, Pthlh, Kl and Fgf23. Among the 389 DEGs, Atoh1, Edn1, Hes1, Kl, Myc, Mycn, Ntf3, Olig1, Ptger4, Tsku and Cxcr4 were associated with inner ear development. Proteins that interacted with Dmp1 and were associated with inner ear development were Opn, Src, Cd4, Hspa5, Lrp5, Fgfr1, Enpp1, Kl, Fgf23 and Mmp9. We wanted to look for the intersection of the three in the Wayne diagram, that is, the DEGs are related with inner ear development and interact with Dmp1. DEGs that met the condition are Klotho genes; at the same time, we found that Fgf23 and Klotho are closely related, and Fgf23 is related with inner ear development and interacts with Dmp1. Finally, we included Fgf23 and Klotho as candidate genes. The Fgf23–Klotho axis, regulated by Dmp1, was hypothesized to be the molecular pathway associated with Dmp1 in inner ear development.

## 4. Discussion

### 4.1. Roles and Studies of Dmp1 in the Mouse Ear 

Previous studies have demonstrated the diverse roles of Dmp1 and its presence in many different types of tissue. In the hearing and balance systems, a recent study reported conductive hearing loss in Dmp1 cKD mice, attributed to a progressive defect in the auditory ossicles [27]. In another study, the involvement of Dmp1 in vestibular bone development was proven through the abnormal circling and head shaking behavior observed in vestibular bone defected mice [28]. It has been found that Dmp1 was present in the inner ear of mice, mainly in the otoconia, the calcium carbonate biominerals involved in the balance function [29,30]. However, there are few studies of Dmp1 expression and function in the membranous labyrinth. In our study, both a specific antibody against Dmp1 and the deficiency of Dmp1 in the OC were used to elucidate the significance of Dmp1 expression in the cochlea. 

### 4.2. Abnormalities in Stereociliary Morphology and a Misorientation of Kinocilia in Dmp1 cKD Mice 

In this study, there was no change in the size of the basilar membrane of the Dmp1 cKD mice, thus excluding a major role for Dmp1 in the regulation of cochlear convergence and extension. However, abnormally flattened stereociliary bundles were more frequently observed in Dmp1 cKD than in WT mice, which suggests that Dmp1 participated in the precise regulation of the hair bundles. The hair bundles act as mechanotransduction sensory receptors in HCs. Their structural characteristics include the graded heights of the stereocilia, forming a V-shaped staircase pattern with the kinocilia, located next to the tallest stereocilia. The asymmetric structure of the hair bundle allows its directional sensitivity to deflection [31,32]. The kinocilium plays a role in the organization of stereociliary bundle morphology [33,34]. The proper emergence and subsequent migration of the kinocilium on the apical surface of HCs are indispensable in the establishment of the short-to-long staircase-like arrangement and the V-shaped orientation of the stereociliary bundles, demonstrated by specifically inactivating ciliary protein genes such as Ift88 and Kif3a [35,36,37]. In our study, the kinocilia were observed by immunohistochemical staining with α-tubulin antibody, with the observed abnormal off-center position confirmed by scanning electron microscopy. As for the mechanisms triggering the centrifugal migration of the kinocilium and the asymmetric shape of the hair bundle, which are still under debate, accumulating evidence links the aberrant localization of the kinocilium to a disorganized microtubule network on the cell surface [38]. The pulling force provided by microtubules connected to the cell cortex is essential for the lateral migration of the kinocilium and for the docking of these structures at their final position [39,40]. Unfortunately, we were unable to investigate whether the microtubule system was disturbed in Dmp1 cKD mice, due to the lack of a specific antibody. Overall, our study suggested that Dmp1 is required for hair bundle morphogenesis; Dmp1 deficiency would lead to the flattening of the stereociliary bundles and the misorientation of kinocilia during the early phase of HC development.

### 4.3. Dmp1 Deficiency Affects the Cell-Intrinsic Polarity of HCs

Auditory HCs display both cellular and tissue-level polarity. In Dmp1 cKD HCs, the flat stereociliary bundles together with the mispositioned kinocilia suggested a defect in cell-intrinsic polarity. However, the gross alignment of hair bundles across the tissue was largely intact, despite the small number of stereocilia in a reversed orientation. Support for the absence of an effect of Dmp1 knockdown on adjacent tissue cell polarity was obtained by Vangl2 staining of the OC in P1 mice. Vangl2, a core PCP protein, is required for the tissue-level PCP to be established in the cochlea [41]. Our results indicated that tissue-level PCP and cell-intrinsic polarity are genetically separable and function independently. PCP signaling is not necessary for the intrinsic planar polarity in individual HCs. In previous experiments, a normal planar polarization and a largely intact apical morphology in individual HCs were retained even in the most severe PCP mutants [42,43]. Further investigation showed that in stereocilia mutants, such as Ift88 or Kif3a cKD mice, core PCP proteins remained asymmetrically localized, despite HC polarization defects, such as in hair bundle orientation, as well as shape defects, kinocilium loss and basal body mispositioning [35,36]. Those studies showed that additional pathways may underlie the establishment of cell-intrinsic polarity in a cell-autonomous fashion.

Several signaling modules that guide the cell-intrinsic molecular blueprint determining hair bundle shape and the planar asymmetry of HCs have been identified, including the Par3-Rac1-Pak, Cdc42-aPKC-Par6 and Insc-Gpsm2-Gαi modules [44,45,46]. Although the interactions between these cell-intrinsic pathways are not fully understood, the formation of a positive feedback loop has been proposed. Par3 and aPKC are asymmetrically localized in the apical cortex, where they recruit the bridging protein Inscuteable (Insc), which in turn binds to its partner (Pins) and the heterotrimeric G protein Gαi. Together, these proteins recruit the effectors needed to generate the pulling forces that cause the astral microtubule to locate to the mitotic spindle. Thus, to characterize the cell-intrinsic polarity change in Dmp1 cKD mice, we focused on Gαi3 and Par6b as representatives of the Gαi/mInsc/LGN and aPKC-Par6 complexes, respectively, and examined whether their asymmetric distributions were abnormal in HCs of Dmp1 cKD mice. Gαi3 is asymmetrically segregated and assumes a crescent shape lateral to the stereociliary bundle at the apical surface of HCs. Opposite Gai3 is a complementary, medial domain of aPKC-Par6b. Gαi3 is thought to be the driving force for the centrifugal migration of the basal body, allowing localization of the kinocilium to the apical surface of HCs [47,48]. We found that, in the deficiency of Dmp1, kinociliary mislocalization was coupled to the expansion of the Gαi3 domain. In addition, the crescents were flatter than those of WT mice, consistent with the flattened stereocilia bundles in the Dmp1 cKD mice. Par6 is a PDZ domain-containing protein that serves as a scaffold for binding and regulating the kinase aPKC, and the tripartite complex formed by Par6, Par3 and aPKC define the apical domain. It thus plays a crucial role in directed migration and the apical–basal polarization of epithelial cells [49,50,51]. Par6 is planar polarized on the medial side whereas in Dmp1 cKD mice the loss of this asymmetry involved the third row of the OHCs. According to these observations, in Dmp1-deficient cochlea, cell-intrinsic polarity was abnormal but the PCP pathway was mostly intact. This would suggest that Dmp1 deficiency eliminates one or more proteins involved in cell-intrinsic molecular anchoring. More studies are needed to investigate the molecular mechanisms of Dmp1 in the maintenance of the cell-intrinsic polarity of HCs.

### 4.4. Transcriptomic Changes in the Cochlea of Dmp1 cKD Mice

To identify pathways potentially altered by Dmp1 deficiency in the cochlea, RNA-seq analysis was performed using cochlear tissue harvested from P1 Dmp1 cKD and WT mice. Only a fraction of the genes in the cochlear transcriptome were differentially expressed. Of these, genes related to transcriptional regulation, cell cycle, DNA repair/maintenance and autophagy were significantly enriched in Dmp1 cKD mice, as were the HIF-1, NF-kappa B and TNF signaling pathways. Many of these pathways have been studied in the inner ear, where they are involved in sensorineural degeneration, HC apoptosis and inflammatory injury [52,53,54]. Our results further suggested their participation in inner ear development. Moreover, the inclusion among the DEGs of genes involved in inner ear development and deafness (Atoh1, Edn1, Hes1, Kl, Myc, Mycn and Olig1) suggested their interaction with Dmp1 in these processes. The DEGs also included Kl and Fgf23, both of which were associated with inner ear development and interact with Dmp1. These genes may be regulated by Dmp1 during inner ear development.

The Fgf23–Klotho endocrine axis is a key modulator of mineral metabolism [55]. Fgf23 regulates phosphate homeostasis as a member of the fibroblast growth factor (FGF) family [56]. Klotho (Kl) was originally identified in connection with premature aging. As a coreceptor for Fgf23 signaling, it increases the affinity of the FGF receptor for Fgf23 and reduces the affinity of the receptor for other FGFs [57,58]. Dmp1 has been shown to suppress Fgf23 expression in bone, most likely through indirect mechanisms [59]. A majority of Klotho protein is expressed in the spiral ligament and stria vascularis of the inner ear and in the kidney tubules [60]. Fgf23 is also broadly expressed throughout the cochlea [61]. Based on the molecular and genetic pathways shared in ear and kidney development, the multiple functions of FGFs in auditory development and the hearing loss phenotype in Klotho KO mice, we hypothesized that Dmp1 may play roles in the auditory system via Fgf23–Klotho axis. Future studies will test this hypothesis using cellular and animal models.

### 4.5. The Normal Hearing Function in Adult Dmp1 cKD Mice

As normal stereociliary bundles are thought to be required for hearing, we assessed the hearing function by measuring auditory brainstem response (ABR) thresholds in Dmp1 cKO and control mice at 4 weeks of age. Surprisingly, no significant difference in hearing sensitivity were observed. Subsequent confocal microscope images and SEM images showed the normal HCs (no HC loss) and normal stereocilia structure. Therefore, we speculate that one possibility is that during the inner ear development, there is a critical period during which planar polarity defects are amended to some extent, as has been reported in the Vangl2 CKO mutants [62]. Dmp1 likely plays a role during a narrow time window in the earlier inner ear developmental stage, and there are compensatory mechanisms in the later stage. The change in Dmp1 protein expression pattern throughout the development seems to support this hypothesis. At P1, the Dmp1 expression coincides with the whole stereociliary bundles and is relatively widespread. By P14, Dmp1 is progressively confined and formed a cloud-shape cluster. This circumscribed expression in the later developmental stage indicates that the influence of Dmp1 is gradually decreasing as hair cells mature. In addition, in the Dmp1 mutant mice, the major phenotype we observed was a flattened stereociliary pattern with a normal stereociliary orientation. Compared to the deficiency of the bundle structure or the disordered stereociliary orientation reported in other mutants, this phenotype is comparably mild. Of course, we only focused on the phenotypic alterations in P1 day mice, which is a limitation of the study. Perhaps we should obverse the hair bundle morphology at different periods (p7, p14, p21, etc.) to find the time point at which the hair bundle defects become amended. In addition, the loss of Dmp1 in conditional mutants may not have physiological impacts until later in life and cause the early onset of age-related hearing loss (the ages of two probands with ARHP owing to a novel homozygous DMP1 mutation and hearing deficit are both over 60 years old). After all, we only measured hearing in mice at 4 weeks of age; these hypotheses could be tested in the future by studying mice at more mature time points.

## 5. Conclusions

Our findings demonstrated a new role of Dmp1 in inner ear development in mice, through the precisely regulated maturation of hair bundles in the auditory epithelium. We showed that Dmp1 is highly expressed in the HCs of the mouse inner ear, where its deficiency leads to stereociliary disorganization and mispositioning of the kinocilia. These findings suggested that Dmp1 plays a role in the precise regulation of hair bundle morphogenesis, by affecting the localization of kinocilia and cell-intrinsic polarity. Moreover, we favored a hypothesis that Dmp1 regulated the kinocilium–stereocilia interaction via the Fgf23–Klotho signaling axis by using RNA-seq analysis, while the molecular targets of Dmp1 remain unknown and await further investigation. In brief, this study shed new light on the Dmp1′s novel function in developing auditory hair cells.

## Figures and Tables

**Table 1:** Primers lists.

Gene	Forward Primer (5′-3′)	Reverse Primer (5′-3′)
Cxcr4	CCATGGAACCGATCAGTGTGAGTA	TTGTCCGTCATGCTCCTTAGCTTC
Wnt3	CACAACACGAGGACGGAGA	AATCTACCCCTTCCCAGTGC
Atoh1	GTA AGG AGA AGC GGC TGT	AGC CAA GCT CGT CCA CTA
Hes1	CCAGCCAGTGTCAACACGA	AATGCCGGGAGCTATCTTTCT
Ptger4	ACCATTCCTAGATCGAACCGT	CACCACCCCGAAGATGAACAT
Gapdh	GCAAGGACACTGAGCAAGA	GGATGGAAATTGTGAGGGAG

**Table 2:** Top 30 up- or downregulated DEGs.

Gene Symbol	log2FoldChange	*p* Value	FDR	Up or Down
Gpha2	3.731	0.00002	0.009	Down
Actc1	3.702	0.00002	0.010	Down
Olig2	3.612	0.00002	0.010	Up
Gm3558	3.561	0.00004	0.016	Down
Lgals1-ps2	3.465	0.00016	0.035	Down
Gdf1	2.874	0.00003	0.011	Up
Il11ra2	2.433	0.00000	0.000	Up
Atoh1	2.342	0.00003	0.013	Down
Oasl1	2.266	0.00019	0.039	Down
Eid3	2.054	0.00001	0.006	Up
Bdkrb2	2.011	0.00000	0.003	Down
Cxcr4	2.010	0.00000	0.000	Down
Sox18	2.000	0.00001	0.005	Down
Slc16a3	1.782	0.00007	0.020	Down
Ptger4	1.776	0.00000	0.003	Down
Ifit3	1.725	0.00016	0.035	Down
Zfp599	1.685	0.00005	0.016	Down
Clec2d	1.640	0.00000	0.002	Down
Hes1	1.549	0.00010	0.025	Down
Zbtb4	1.537	0.00000	0.000	Up
Cnih3	1.529	0.00025	0.047	Up
Cited2	1.527	0.00000	0.000	Down
Elf3	1.430	0.00022	0.042	Down
Ntf3	1.371	0.00001	0.005	Down
Fzd8	1.342	0.00004	0.016	Down
Myc	1.293	0.00000	0.000	Down
Ccnd3	1.151	0.00001	0.006	Down
Rtp4	1.098	0.00016	0.035	Down
Zfp84	1.097	0.00003	0.014	Down
Wnt3	1.096	0.00007	0.020	Down

## Data Availability

Not applicable.

## Supplementary Materials

The following supporting information can be downloaded at https://www.mdpi.com/article/10.3390/biology12040625/s1, Figure S1: β-catenin, β-spectrin and ZO-1 localization. Figure S2: Heatmap generated by clustering 389 DEGs and 6 samples together. Figure S3: The normal hearing and normal hair cells in adult Dmp1 cKD mice. Figure S4: 1st-dmp1. Figure S5: 1st-gapdh. Figure S6: 2st-dmp1. Figure S7: 2st-gapdh. Figure S8: 3st-dmp1. Figure S9: 3st-gapdh.
