# Supplementary material for: Involvement of Dmp1 in the Precise Regulation of Hair Bundle Formation in the Developing Cochlea
Source: Biology (Basel). 2023 Apr 20;12(4):625. doi: 10.3390/biology12040625 (PMC10135853; doi:10.3390/biology12040625)
Supplement: Supplementary file 1 [file biology-12-00625-s001.zip › Figures S1-S3.pdf]

Supplementary Materials

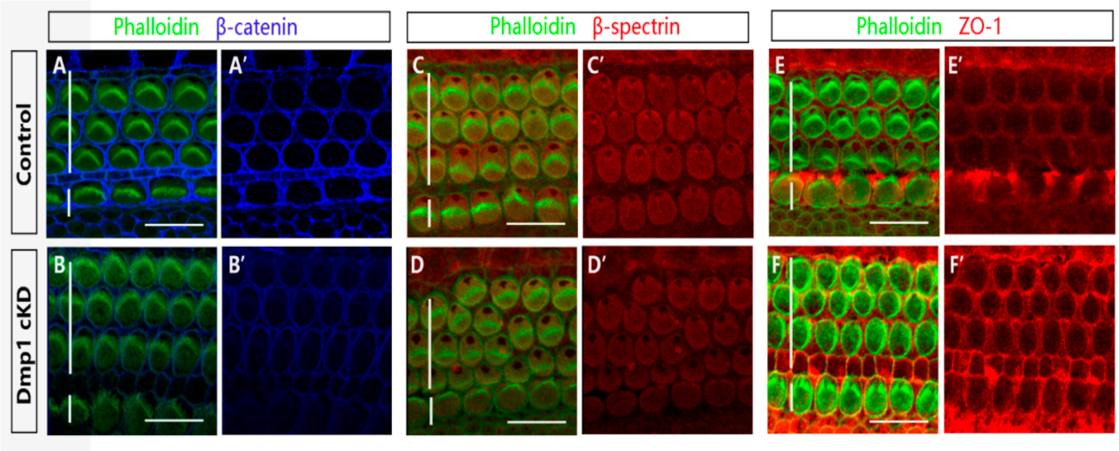

Figure S1. Catenin (A,B), β-spectrin (C,D) and ZO-1 (E,F) localization in the control and Dmp1 cKD OC at P1. Cytoskeleton and junctions were marked by phalloidin staining. The expression of these proteins was not significantly altered. Scale bars: 10 μm.

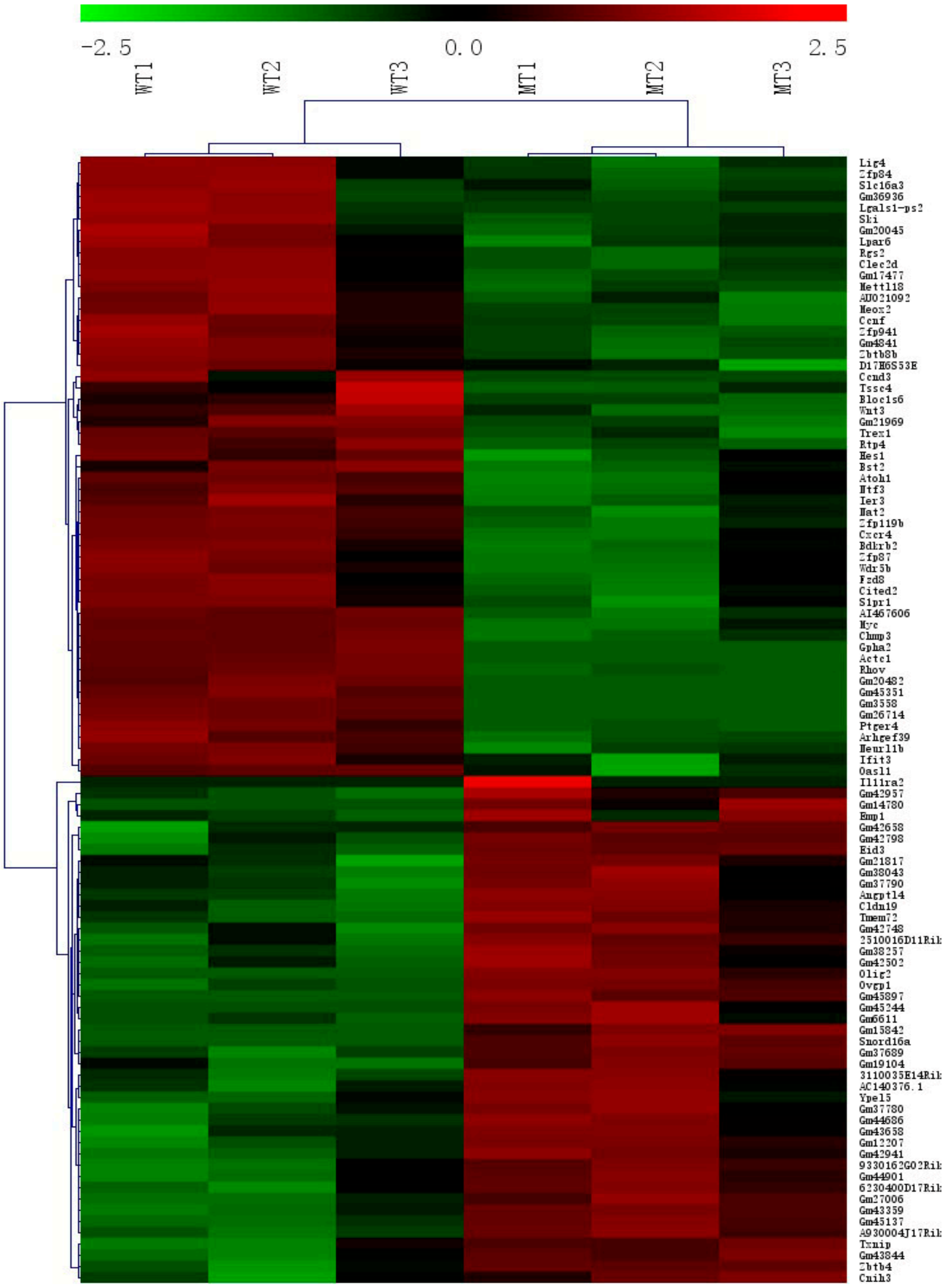

Figure S2. Heatmap generated by clustering 389 DEGs and 6 samples together.

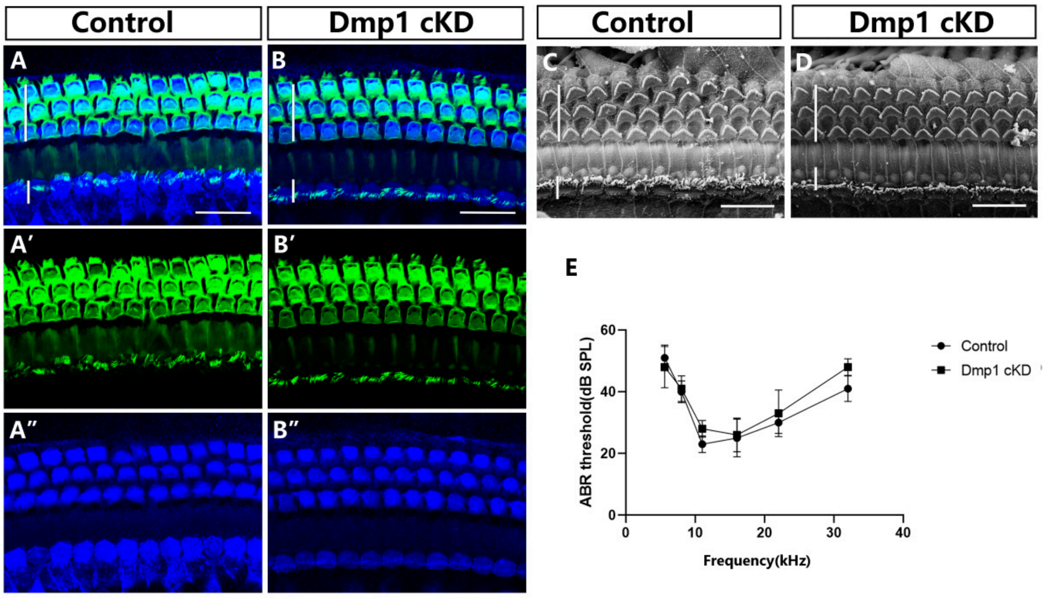

Figure S3. The normal hearing and normal hair cells in adult Dmp1 cKD mice. (A,B):Whole-mount images of adult cochlea from control and Dmp1 cKD mice. There was no loss of hair cells in Dmp1 cKD cochlea. (C,D): SEM images of adult cochlea from control and Dmp1 cKD mice. The hair bundles were nomal. (E): ABR thresholds of 4-week-old Dmp1 cKD mice (n = 5) and controls (n = 6). No significant difference in hearing sensitivity was observed.
