# Supplementary figures and images for: Involvement of Dmp1 in the Precise Regulation of Hair Bundle Formation in the Developing Cochlea
Source: Biology (Basel). 2023 Apr 20;12(4):625. doi: 10.3390/biology12040625 (PMC10135853; doi:10.3390/biology12040625)

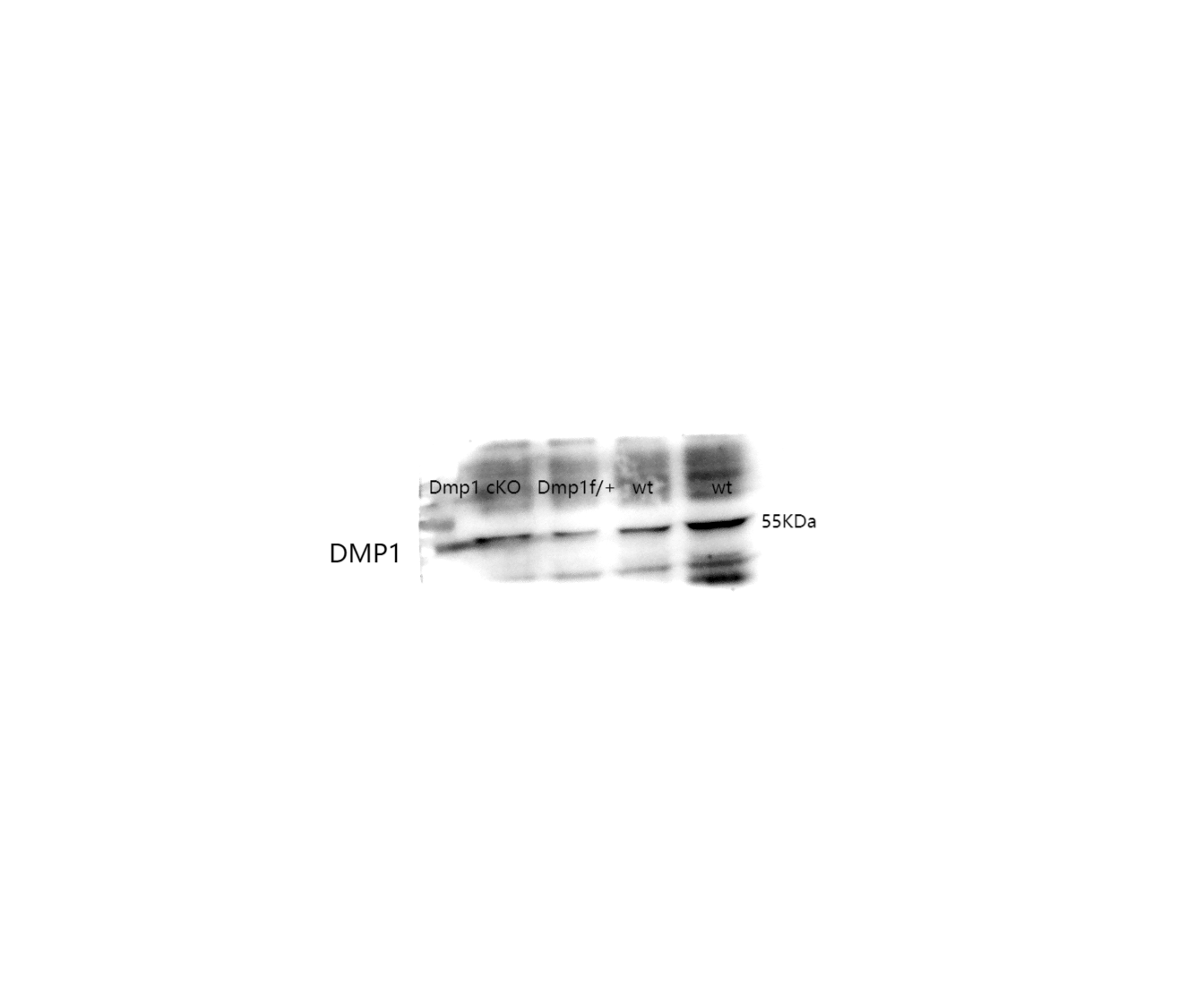

Supplement: Supplementary file 1 [file biology-12-00625-s001.zip › Figure S4. 1st-dmp1.png]

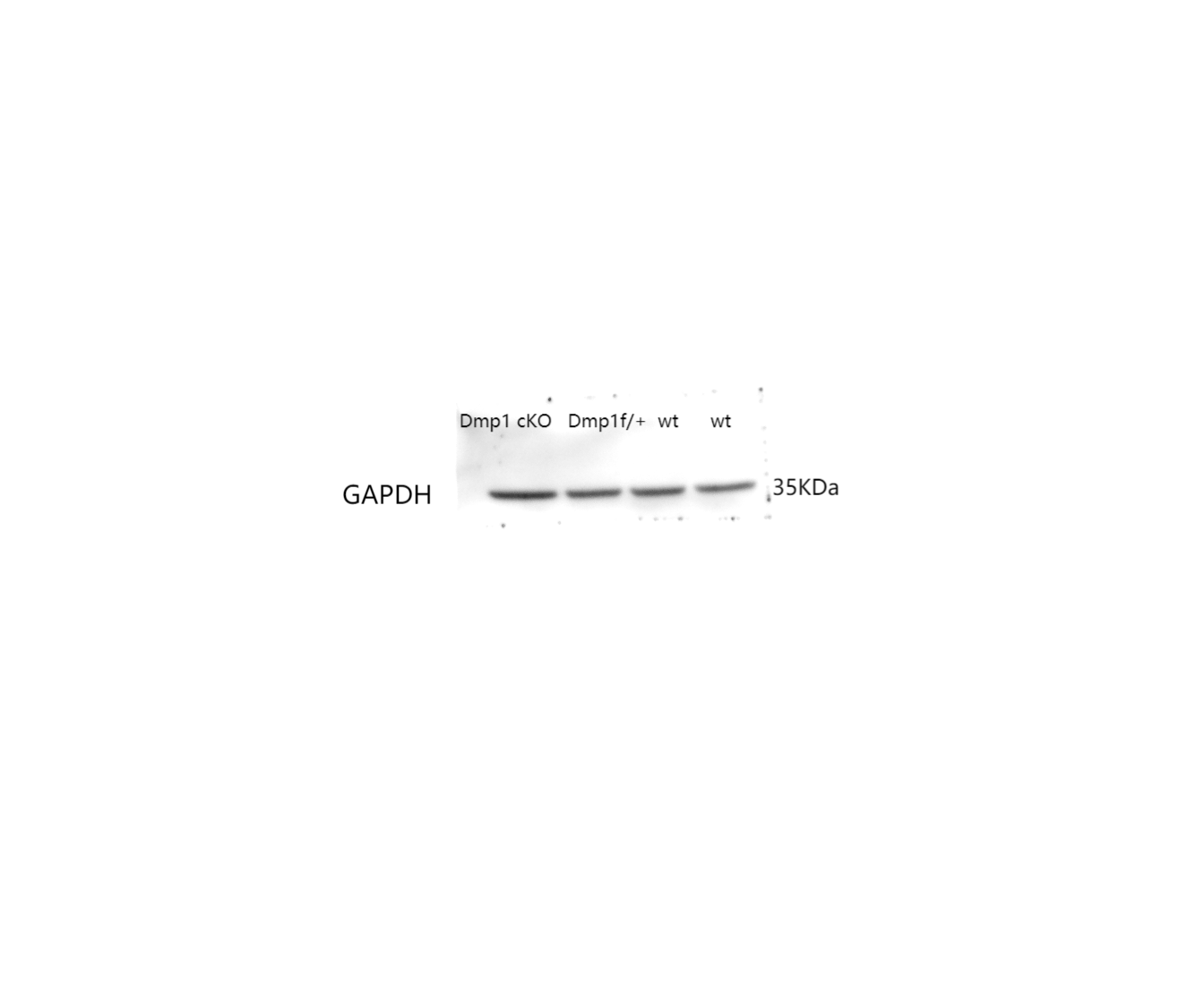

Supplement: Supplementary file 1 [file biology-12-00625-s001.zip › Figure S5. 1st-gapdh.png]

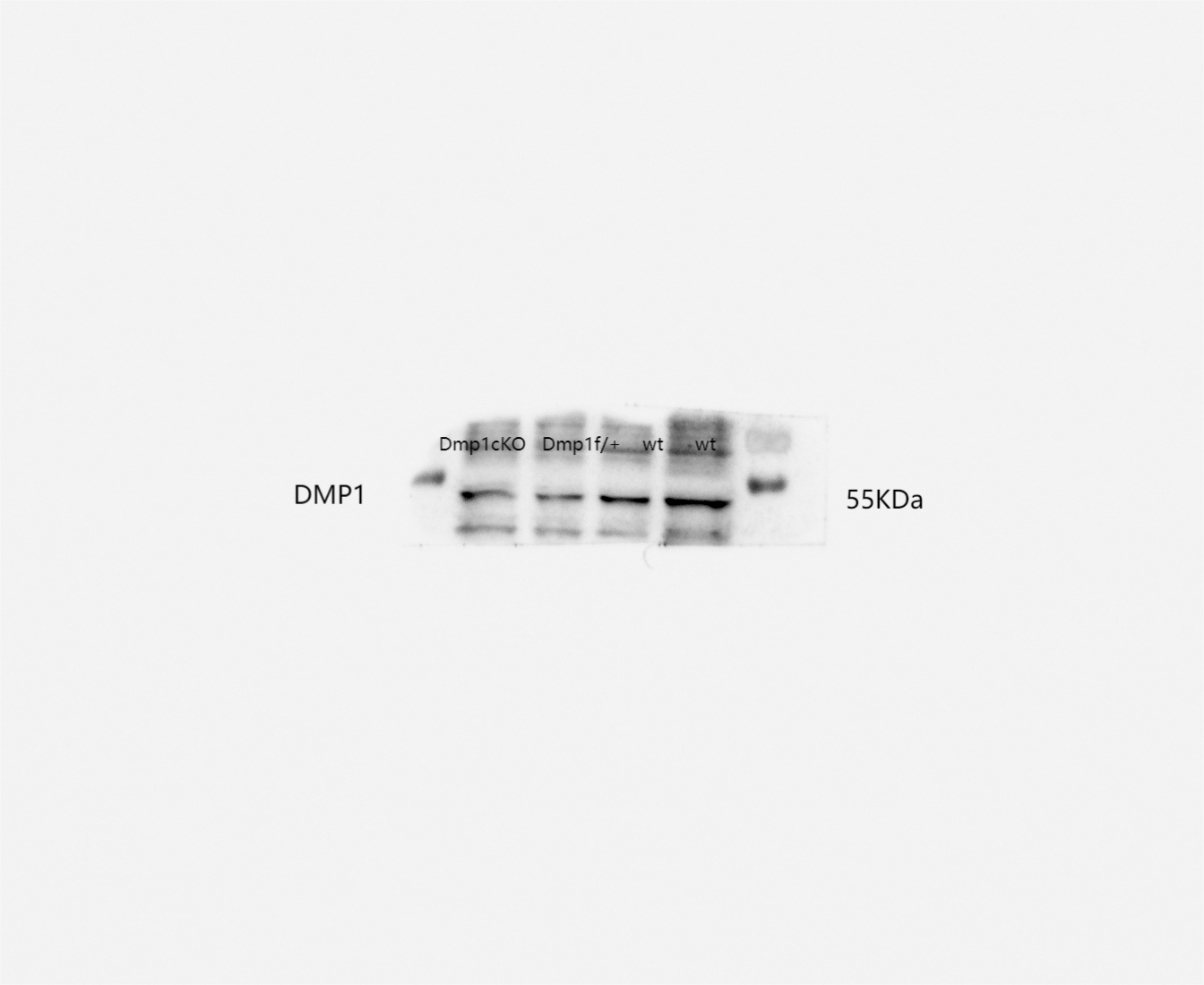

Supplement: Supplementary file 1 [file biology-12-00625-s001.zip › Figure S6. 2st-dmp1.png]

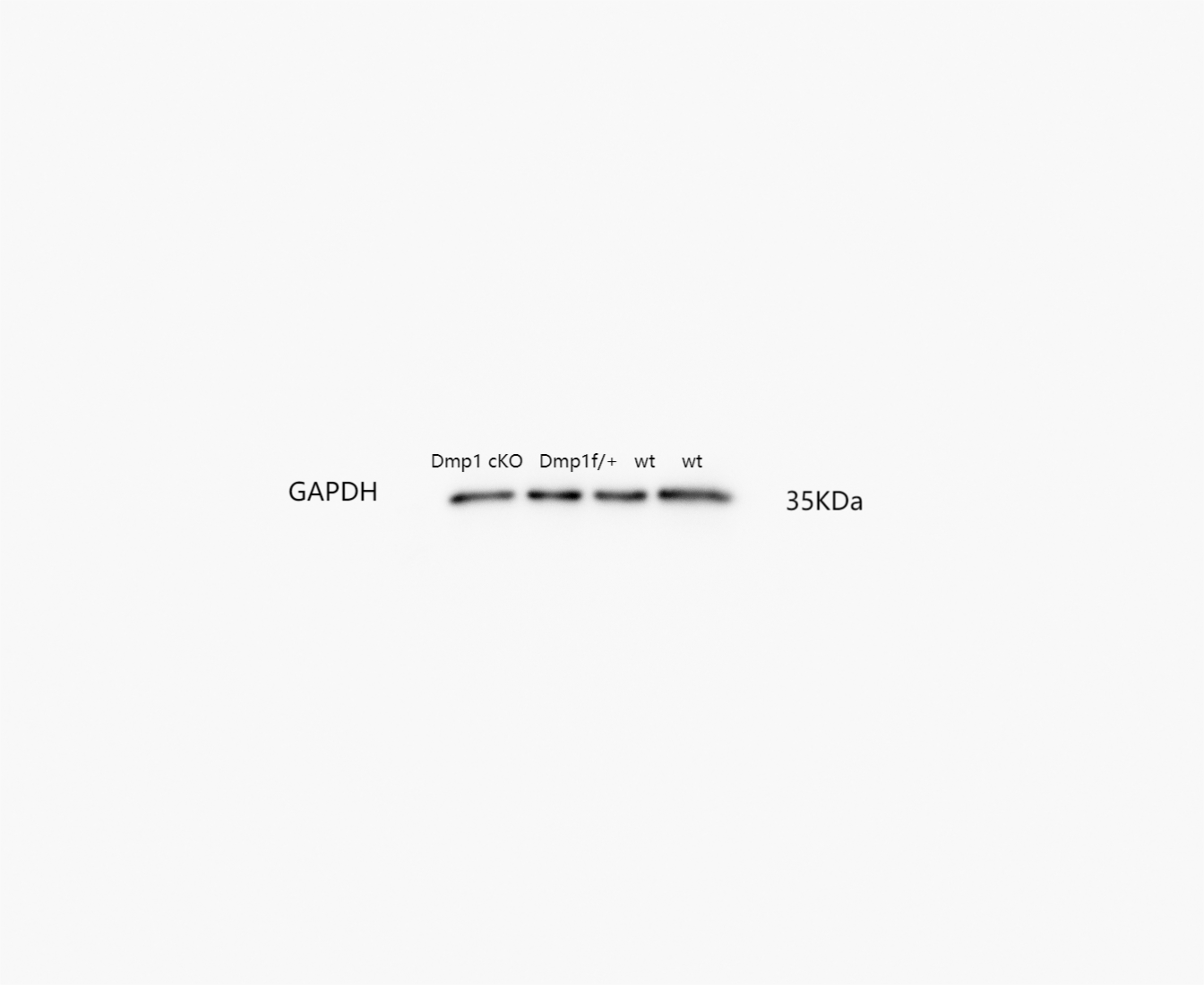

Supplement: Supplementary file 1 [file biology-12-00625-s001.zip › Figure S7. 2st-gapdh.png]

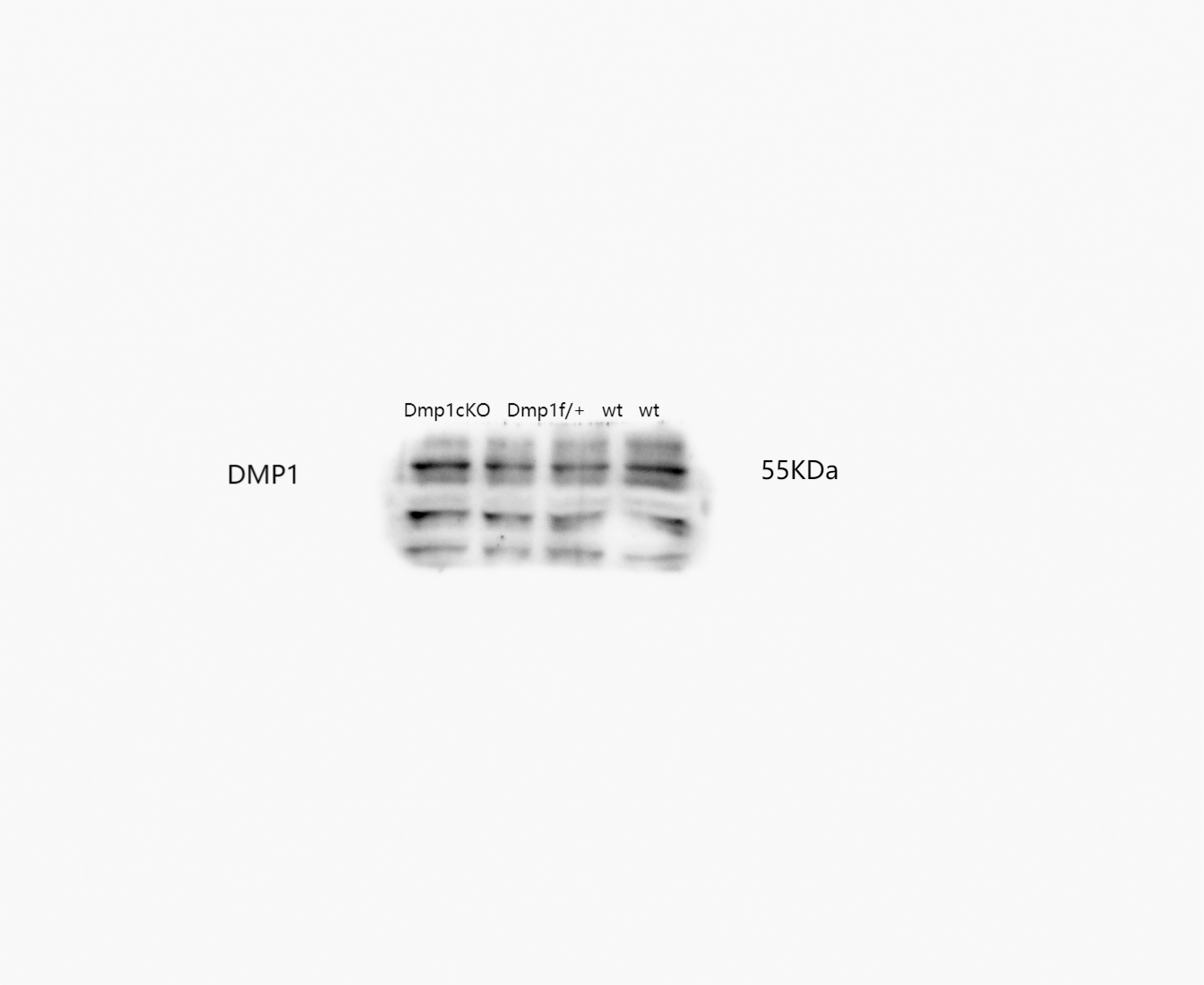

Supplement: Supplementary file 1 [file biology-12-00625-s001.zip › Figure S8. 3st-dmp1.png]

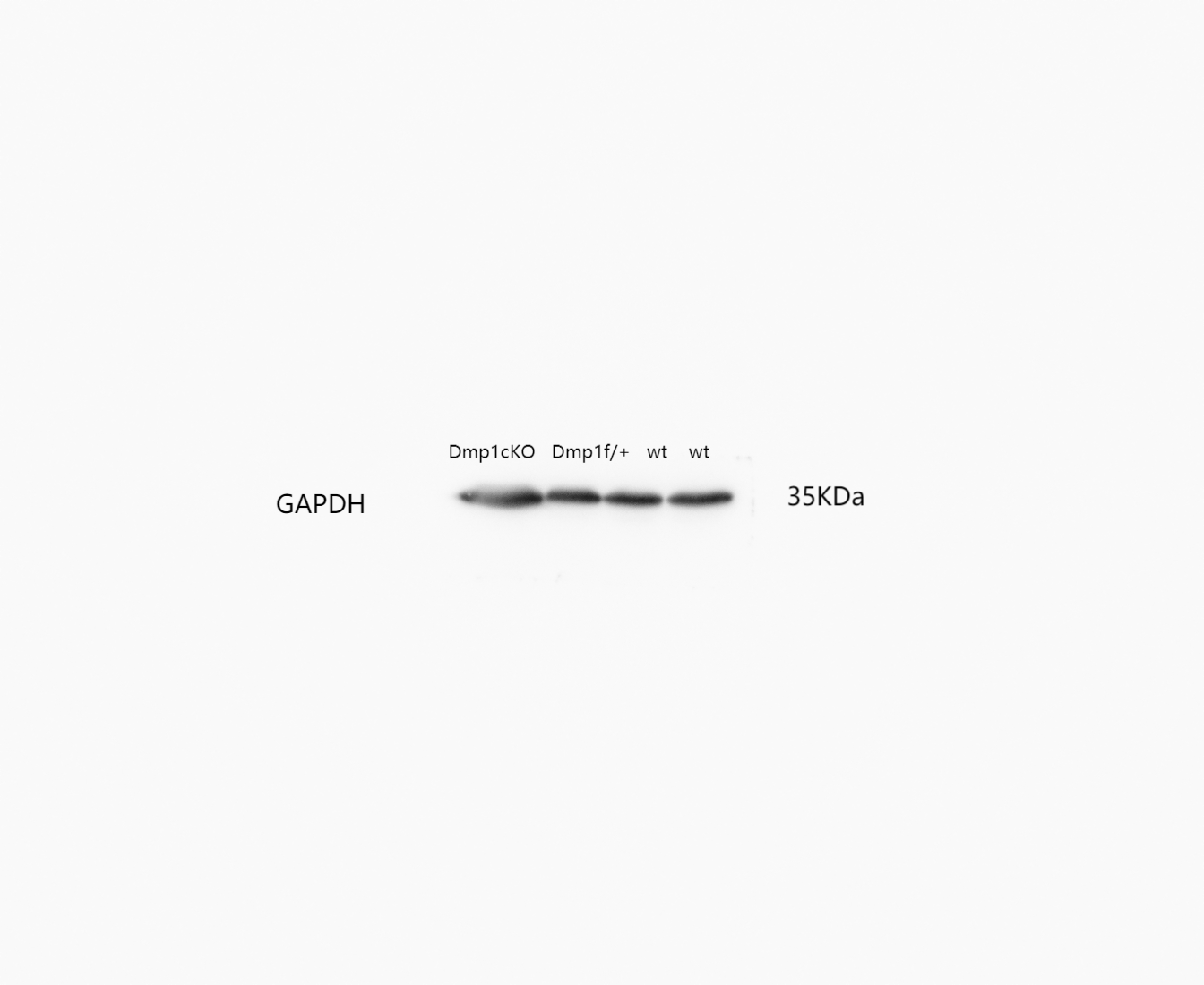

Supplement: Supplementary file 1 [file biology-12-00625-s001.zip › Figure S9. 3st-gapdh.png]
